# A New 4α-Methylated Sterol from a *Nephthea* sp. (Nephtheidae) Bornean Soft Coral 

**DOI:** 10.3390/molecules14093360

**Published:** 2009-09-02

**Authors:** Takahiro Ishii, Hiroshi Matsuura, Zhan Zhaoqi, Charles Santhanaraju Vairappan

**Affiliations:** 1 Laboratory of Natural Products Chemistry, Institute for Tropical Biology and Conservation, Universiti Malaysia Sabah, 88999 Kota Kinabalu, Sabah, Malaysia; E-mail: ishii_t@ums.edu.my (T.I.); 2 Graduate School of Environmental Science, Hokkaido University, Sapporo 060-0810, Japan; E-mail: matsuura@ees.hokudai.ac.jp (H.M.); 3 Shimadzu (Asia Pacific) Pte Ltd, 16 Science Park Drive, #01-01, The Pasteur Singapore Science Park, 118227 Singapore; E-mail: zhaoqi@shimadzu.com.sg (Z.Z.)

**Keywords:** 4α-methyl steroid, *Nephthea* sp., Nephtheidae, soft coral

## Abstract

A new 4α-methyl sterol, 4α-methyl-ergosta-6,8(14),22*E*-triene-3β-ol (**1**), was isolated along with cholesterol from a *Nephthea* sp. Bornean soft coral. The structure of compound **1** was elucidated on the basis of spectroscopic analysis and comparison of the data with those of the related compounds.

## Introduction

Marine organisms constitute a rich source of diverse and complex sterols; particularly among marine invertebrates the complexity of sterols arises through food chains and symbiotic relationships between organisms [1]. It has been observed that 4α-methyl steroids are often end products of steroids biosynthesis in the dinoflagellates and intermediates in steroids biosynthesis in animals and in other divisions of the Plant Kingdom [2,3]. Previous chemical investigations on soft coral have identified a variety of 4α-methyl sterols, possibly synthesized by the dinoflagellate symbiont of the soft coral [4,5,6,7,8,9]. The family Nephtheidae comprises many genera, among which *Lemnalia*, *Paralemnalia*, *Capnella*, *Lithophyton*, *Dendronephthya*, *Scleronephthya*, *Stereonephthya* and *Nephthea* have received considerable attention from organic chemists [10]. Among Octocorallia the genus *Nephthea* comprises a large variety of species. A literature search revealed that the genus *Nephthea* has afforded a variety of sesquiterpenes, diterpenes and steroids [4,5,6,11,12,13,14,15,16,17,18,19], but there have been no reports on chemical constituents of Malaysian soft corals. We have now examined an unidentified specimen collected from Sepanggar Island (Sabah, Malaysia), whose methanol extract afforded a new 4α-methyl sterol, identified as 4α-methyl-ergosta-6,8(14),22*E*-triene-3β-ol (**1**) and cholesterol (**2**). In this paper we report the isolation and structural determination by spectroscopic methods of new compound **1**.

**Figure 1:**
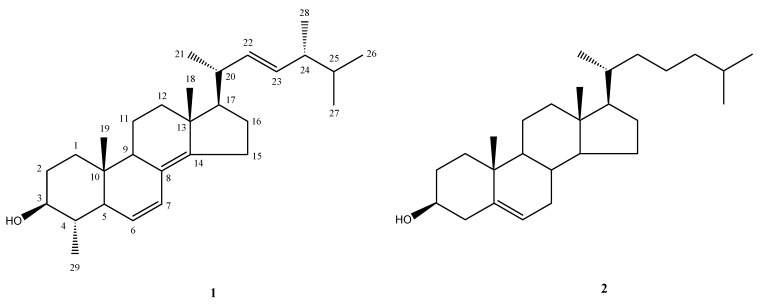
Structures of compounds **1** and **2**.

## Results and Discussion

The sample was collected from Sepanggar Island (Sabah, Malaysia) and extracted with MeOH. The MeOH extract was concentrated and subsequently subjected to further purification to yield a new 4α-methyl sterol **1** and the known compound **2**. Compound **2** was identified as cholesterol by comparing its spectral data with those reported in the literature [20].

Compound **1** was isolated as a white amorphous solid. HR-MS established a molecular formula of C_29_H_46_O, implying seven degrees of unsaturation. The ^1^H-NMR spectrum of **1** clearly showed seven methyl signals at δ_H_ 0.65 (3H, s, H-19), δ_H_ 0.81 (3H, d, *J* = 6.9 Hz, H-27), δ_H_ 0.83 (3H, d, *J* = 6.9 Hz, H-26), δ_H_ 0.89 (3H, s, H-18), δ_H_ 0.91 (3H, d, *J* = 6.9 Hz, H-28), δ_H_ 1.02 (3H, d, *J* = 6.2 Hz, H-21) and δ_H_ 1.09 (3H, d, *J* = 6.2 Hz, H-29), four trisubstituted olefinic protons at δ_H_ 5.18 (1H, dd, *J* = 15.1, 8.3 Hz, H-22), δ_H_ 5.22 (1H, dd, *J* = 15.1, 6.9 Hz, H-23), δ_H_ 5.60 (1H, d, *J* = 10.3 Hz, H-7) and δ_H_ 6.15 (1H, dd, *J* = 10.3, 2.8 Hz, H-6), and one oxymethine proton at δ_H_ 3.15 (1H, m, H-3). The proton at δ_H_ 3.15 suggested the existence of the characteristic hydroxyl group at C-3 of 4α-methyl steroids [4]. The ^13^C-NMR and DEPT spectra of **1** also exhibited seven methyl carbons [δ_C _21.2 (q, C-21), 20.0 (q, C-26), 19.7 (q, C-27), 19.5 (q, C-18), 17.7 (q, C-28), 15.1 (q, C-29) and 12.4 (q, C-19),], six olefinic carbons [δ_C_ 147.3 (s, C-14), 135.5 (d, C-22), 132.2 (d, C-23), 126.2 (d, C-6), 126.0 (d, C-7) and 125.0 (s, C-8)] and one OH-bearing carbon (δ_C_ 77.3, d, C-3). The NMR and HRMS data could thus account for three of the seven degrees of unsaturation, suggesting the tetracyclic nature of **1**.

**Figure 2:**
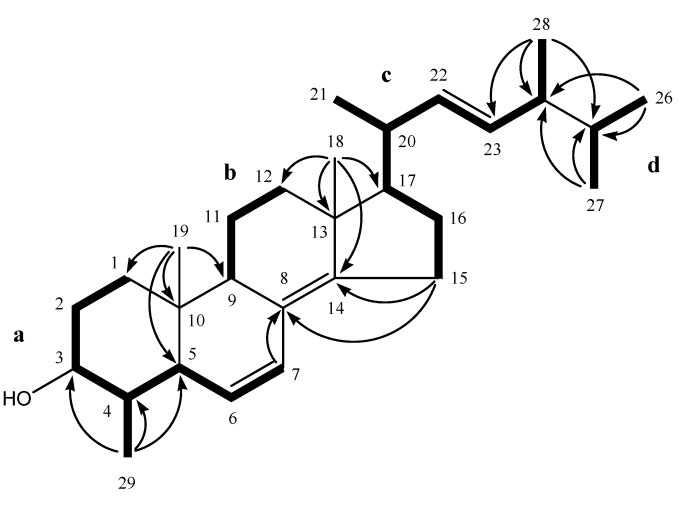
^1^H-^1^H COSY correlations (bold lines) and key HMBC correlations (H → C) of **1**.

All C–H correlations of **1** were detected in the HSQC experiment. The ^1^H–^1^H COSY spectrum exhibited partial structures **a**, **b**, **c** and **d** (Figure 2). Confirmation of the partial structures and their connectivity was made with the aid of the HMBC spectrum. HMBC correlations between H_3_-19 and C-1/C-5/C-9/C-10 established partial structure **a,** could be connected to **b** through a quaternary carbon (C-10). HMBC correlations between H_3_-18 and C-12/C-13/C-17 revealed partial structure **b** could be connected to **c** through a quaternary carbon (C-13). Furthermore, HMBC correlations between H_3_-26/C-24, H_3_-27/C-24 and H_3_-28/C25 established the connection of partial structure **c** with **d**. Partial structures **a **and **c** were connected through fully substituted double bond between C-8 and C-14 by HMBC cross-peaks between H-7/C-8, H_2_-15/C-8, H_2_-15/C-14 and H_3_-18/C-14. Based on available spectroscopic data obtained for this compound, there were no other available connection option then of C-8 to C-9. HMBC correlations from H_3_-29 to C-3, C-4 and C-5 confirmed the existence of a 4α-methyl group. Based on these findings, the planar structure of **1** was concluded as shown in Figure 1.

**Figure 3:**
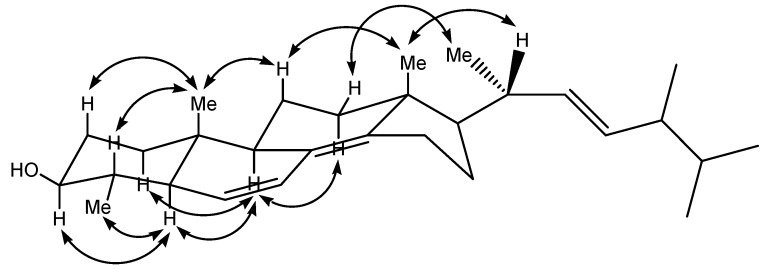
Key NOESY correlations of **1**.

The relative stereochemistry of compound **1** was deduced from the NOESY experiment as well as the coupling constants in the ^1^H-NMR spectrum. The coupling constant between H-22 and H-23 (*J* = 15.1 Hz) suggested the double bond to have *E* configuration. Furthermore, as shown in Figure 3, the NOESY correlations observed between H-1α/H-9, H-2β/H_3_-19, H-3/H-5, H-4/H_3_-19, H-5/H-9, H-5/H_3_-29, H-9/H-12α, H-11β/H_3_-18, H-11β/H_3_-19, H-12β/H_3_-21 and H_3_-18/H-20 revealed the relative configurations for each ring junction and chiral center. The configuration at C-24 was proposed by comparison of its NMR data with those of model compounds (Table 2) [21,22]. Thus, compound **1** was identified as 4α-methyl-ergosta-6,8(14),22*E*-triene-3β-ol.

**Table 1:** ^1^H NMR and ^13^C NMR spectral data of compound **1** (recorded at 600/150 MHz in CDCl_3_; δ in ppm, *J* in Hz).

Position	^13^C	^1^H (*J* in Hz)
1	35.1 (CH_2_)	1.69 (m, 1H)
		1.17 (m, 1H)
2	31.2 (CH_2_)	1.88 (m, 1H)
		1.54 (m, 1H)
3	77.3 (CH)	3.15 (m, 1H)
4	38.1 (CH)	1.38 (m, 1H)
5	51.2 (CH)	1.69 (dd, *J* = 9.6, 2.8 Hz, 1H)
6	126.2 (CH)	6.15 (dd, *J* = 10.3, 2.8 Hz, 1H)
7	126.0 (CH)	5.60 (d, *J* = 10.3 Hz, 1H)
8	125.0 (C)	
9	48.5 (CH)	1.92 (m, 1H)
10	36.4 (C)	
11	19.7 (CH_2_)	1.60 (m, 1H)
		1.45 (m, 1H)
12	36.8 (CH_2_)	1.98 (ddd, *J* = 12.4, 3.5, 3.5 Hz, 2H)
		1.27 (m, 1H)
13	43.5 (C)	
14	147.3 (C)	
15	25.0 (CH_2_)	2.35 (m, 1H)
		2.27 (m, 1H)
16	28.0 (CH_2_)	1.75 (m, 1H)
		1.40 (m, 1H)
17	56.1 (CH)	1.19 (m, 1H)
18	19.5 (CH_3_)	0.89 (s, 3H)
19	12.4 (CH_3_)	0.65 (s, 3H)
20	39.6 (CH)	2.09 (m, 1H)
21	21.2 (CH_3_)	1.02 (d, *J* = 6.2 Hz, 3H)
22	135.5 (CH)	5.18 (dd, *J* = 15.1, 8.3 Hz, 1H)
23	132.2 (CH)	5.22 (dd, *J* = 15.1, 6.9 Hz, 1H)
24	43.0 (CH)	1.85 (m, 1H)
25	33.2 (CH)	1.46 (m, 1H)
26	20.0 (CH_3_)	0.83 (d, *J* = 6.9 Hz, 3H)
27	19.7 (CH_3_)	0.81 (d, *J* = 6.9 Hz, 3H)
28	17.7 (CH_3_)	0.91 (d, *J* = 6.9 Hz, 3H)
29	15.1 (CH_3_)	1.09 (d, *J* = 6.2 Hz, 3H)

**Table 2:** Partial ^13^C-NMR spectral data of the model compounds (crinosterol and brassicasterol) and **1**.

**Position**	**Crinosterol (24S)**	**Brassicasterol (24R)**	**Compound 1**
	^13^C	^13^C	^13^C
**24**	43.12	42.90	42.95
**25**	33.28	33.16	33.19
**26**	19.69	20.02	20.04
**27**	20.19	19.69	19.73
**28**	18.08	17.68	17.71

## Experimental

### General

Optical rotations were measured on an AUTOPOL IV automatic polarimeter (Rudolph Research Analytical). ^1^H-NMR (600 MHz) and ^13^C-NMR (150 MHz) spectra were recorded with a JEOL ECA 600, with TMS as internal standard. HR-ESI-TOFMS spectrum was obtained with LCMS-IT-TOF (Shimadzu) in ESI mode. HPLC was conducted on a Waters 600 using UV detector, Luna 5μ Phenyl-hexyl (10.0 × 250 mm) and Luna 5μ C18(2) 100A (10.0 × 250 mm). Preparative TLC was performed with silica gel plates (Merck, Kieselgel 60 F_254_). Silica gel (Merck, Kieselgel 60, 70-230 mesh) was used for column chromatography. Analytical TLC was performed on Merck Kieselgel 60 F_254_. Spots were visualized by UV light or by spraying with a 5% phosphomolybdic acid-ethanol solution.

### Biological material

A specimen of *Nephthea* sp. was collected from Sepanggar Island, Sabah (6^o^04’017’’N, 116^o^04’836’’E), on January 24, 2008. The voucher specimen was deposited in the herbarium of Institute for Tropical Biology and Conservation, Universiti Malaysia Sabah (BORNEENSIS).

### Extraction and isolation

The fresh soft coral (400 g wet wt) was extracted with MeOH at room temperature for 7 days. The crude extract was evaporated under reduced pressure and the residue was partitioned between EtOAc and H_2_O. The EtOAc fraction was further partitioned with hexane and 90% MeOH. The hexane fraction (1.0 g) was fractionated by Si gel column chromatography with a step gradient of hexane and EtOAc in the ratio of 9:1, 8:2, 7:3, 1:1 and EtOAc. The fraction (237 mg) eluted with hexane/EtOAc (8:2) was further separated by a combination of preparative TLC with CHCl_3_ and HPLC (Luna 5μ Phenyl-hexyl) with 80% MeCN to afford compound **1** (1.8 mg). The fraction (20 mg) eluted with hexane/EtOAc (7:3) was separated by repeated preparative TLC with CHCl_3_ and hexane/EtOAc (3:1) to give compound **2 **(3.2 mg).

### Characterization of 4α-Methyl-ergosta-6,8(14),22E-triene-3β-ol ***(1)***

White amorphous solid; [α]^25^_D_ -36.9 (*c* 0.13, CHCl_3_); HR-TOFMS *m*/*z* 411.3593 [M+H]^+^ (calcd. for C_29_H_47_O, 411.3621); ^1^H-NMR and ^13^C-NMR spectral data: see Table 1.
